# Metallogels as Hybrid Metal-Organic Soft Materials: Classification, Fabrication Pathways and Functional Applications

**DOI:** 10.3390/gels12020124

**Published:** 2026-02-01

**Authors:** Maciej Grabowski, Tomasz Grygier, Anna Trusek

**Affiliations:** Department of Micro, Nano and Bioprocess Engineering, Wroclaw University of Science and Technology, Wybrzeze Wyspianskiego 27, 50-370 Wroclaw, Poland

**Keywords:** metallogels, metal-organic gels, metal-phenolic networks, nanoparticle gels, supramolecular gels

## Abstract

Metallogels constitute a rapidly expanding class of hybrid soft materials in which metal ions, metal complexes, or metal-containing nanoparticles play a decisive structural and functional role within a three-dimensional gel network. Their unique combination of supramolecular assembly, metal-ligand coordination, and dynamic network behaviour provides tunable mechanical, optical, electrical, redox, and catalytic properties that are not accessible in conventional hydrogels or organogels. This review systematically summarises current knowledge on metallogels, beginning with a classification based on matrix type, dominant metal interaction and functional output, spanning metallohydrogels, metal-organic gels, metal-phenolic gels, nanoparticle-based gels, polymer-based metallogels and low-molecular-weight metallogels. Key synthesis pathways are discussed, including coordination-chemistry-driven formation, metal-ligand self-assembly, in situ reduction, diffusion-mediated strategies, sol-gel-like polymerisation, enzyme-assisted routes, and bio-derived fabrication. Particular emphasis is placed on structure-function relationships that enable the development of catalytic, conductive, luminescent, antimicrobial, and biomedical metallogels. The examples compiled here highlight the versatility and transformative potential of metallogels in next-generation soft technologies, including sensing, energy conversion, wound healing, drug delivery, and emerging applications such as soft electronics and on-skin catalytic or bioactive patches. By mapping current progress and emerging design principles, this review aims to support the rational engineering of metallogels for advanced technological and biomedical applications

## 1. Introduction

The first example of a system that could be called a metallogel dates back to 1841, when A. Lipowitz reported a lithium urate hydrogel [1]. Although Lipowitz’s work predates the concept of metallogels by more than a century, his observations unintentionally touch on a phenomenon that today would be recognised as gel-like behaviour in metal-organic systems. When large amounts of uric acid were dissolved in lithium carbonate solution and then cooled, the mixture formed a reversible gelatinous mass. Modern terminology suggests that such behaviour resembles a proto-metallogel, in which a metal ion (Li^+^) interacts with an organic ligand (uric acid) to form a semi-solid, highly hydrated network. However, unlike true metallogels—which possess a stable, self-assembled coordination network—Lipowitz’s gel was not structurally persistent and easily re-dissolved upon heating. Still, his experiment represents an early empirical observation of how metal ions can induce gelation-like transitions in organic systems. Although such early gel-like phenomena were observed empirically, their interpretation remained largely phenomenological until the development of supramolecular chemistry and coordination-driven self-assembly provided a conceptual framework for understanding and deliberately designing metal-containing gel networks. The term metallogel entered the vocabulary of supramolecular chemistry and materials science in the late 20th to early 21st century, when metal-coordinated gels were recognised as a distinct class of soft materials, driven by increased research into coordination hydrogels, metal-ligand self-assembly, and supramolecular gels.

Metallogels represent an important subclass of supramolecular gels in which metal ions or metal complexes play an integral role in establishing and stabilising the three-dimensional network. The presence of metal centres is not only essential for initiating or enhancing gelation but also enables precise modulation of key gel properties, including morphology, mechanical strength, and resp`onsiveness [2]. Furthermore, incorporating metal-based components provides an effective way to combine the properties of metals with those of the organic matrix. This enables precise modulation of key characteristics such as conductivity, colour, rheological behaviour, adsorption capacity, emission, photophysical properties, magnetism, antibacterial activity, catalytic performance, redox behaviour, and self-healing capability [3,4,5,6]. Consequently, metallogels can exhibit a broad and versatile range of responses to physical and chemical stimuli. For instance, the introduction of multivalent ions such as Co^2+^/^3+^, Cu^2+^/^3+^ or Fe^2+^/^3+^, creates redox-responsive hydrogels, whereas the addition of magnetic nanoparticles such as Fe_3_O_4_ makes them sensitive to external magnetic field [7,8,9,10].

## 2. Classification of Metallogels

**Figure 1:**
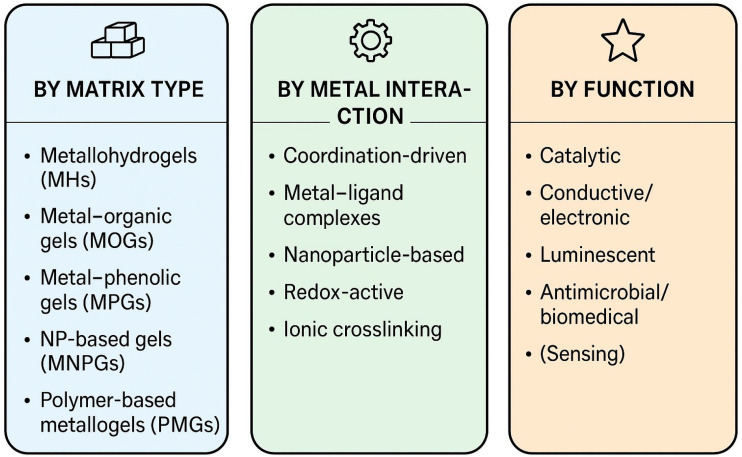
Classification of metallogels.

There are many ways to classify metallogels. They can be divided into matrix type, type of metal interaction, and function. An overview of these classification criteria and their interrelationships is schematically summarised in Figure 1.

It should be noted that the classification schemes presented in this section are not mutually exclusive. For example, a polymer-based metallohydrogel can be classified according to its matrix type (metallohydrogel or polymer-based metallogel), the mechanism by which the metals interact (e.g., coordination-driven or ionically cross-linked), and its functional behaviour (e.g., stimuli-responsive, self-healing or biomedical).

### 2.1. Based on Matrix

This classification of metallogels is based on the nature of the metal-containing matrix, which arises from different types of gelators (e.g., polymers, nanoparticles, polyphenols, small molecules) and their corresponding gelation mechanisms. According to this criterion, we can distinguish several types of metallogel, including Metallohydrogels (MHs), Metal-Organic Gels (MOGs), Metal–Phenolic Gels (MPGs), Metal Nanoparticle-Based Gels (MNPGs), Polymer-Based Metallogels (PMGs), and Low-Molecular-Weight Metallogels (LMW-MGs) [11,12,13].

#### 2.1.1. Metallohydrogel (MHs)

**Figure 2:**
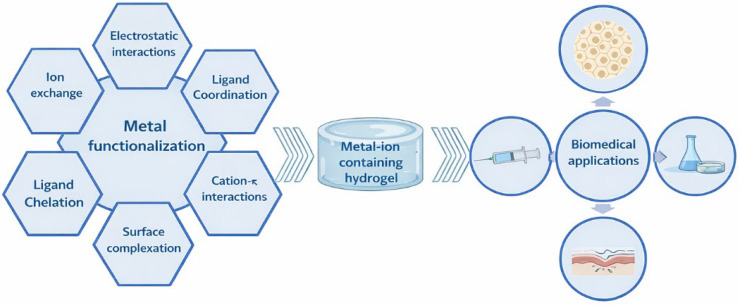
Schematic illustration of the primary mechanisms governing the formation and functionalization of metal-ion-containing hydrogels. Metal incorporation occurs via multiple non-covalent and coordination interactions—including ion exchange, electrostatic attraction, hydrogen bonding, surface complexation, cation–π interactions, and coordination/chelation—that collectively drive network assembly and stabilisation. The resulting metallohydrogels possess tunable physicochemical and mechanical properties enabling diverse biomedical applications, such as drug delivery, wound healing, and tissue engineering [12].

A metallohydrogel is a type of supramolecular hydrogel in which a three-dimensional, water-rich network is formed or reinforced through the coordination of metal ions and organic ligands [14,15,16,17]. In these systems, metal ions such as Zn^2+^, Cu^2+^, Fe^3+^, Ni^2+^, or Mg^2+^ interact with small-molecule gelators, peptides or polymeric ligands, inducing self-assembly into fibrous or particulate networks that can bind large volumes of water. The coordination bonds that serve as cross-links typically exhibit intermediate binding strengths and dynamic reversibility. This imparts self-healing, shear-thinning and stimuli-responsive behaviour, including sensitivity to pH changes, redox environments, competing ligands, temperature and light [14,16]. The primary mechanisms governing the formation and functionalisation of metallohydrogels are schematically illustrated in Figure 2.

As the structural and physicochemical properties of these hydrogels depend heavily on the identity, valence state, and coordination geometry of the metal ion, metallohydrogels can be widely tuned for mechanical stiffness, viscoelasticity, and network dynamics [14,15]. Their hybrid nature, combining the hydration, softness, and biocompatibility of traditional hydrogels with the catalytic, electronic, and structural functions of metal coordination, enables a diverse range of applications. These include drug delivery and controlled release, tissue engineering and wound healing, chemical and biological sensing, catalysis, and functional soft materials such as conductive or semiconducting gels [14,15,16,18]. Within the broader classification of supramolecular hydrogels, metallohydrogels represent a distinct category in which coordination-driven assembly plays the primary structural role. This distinguishes them from hydrogel systems driven by hydrogen bonds, π–π stacking, or host–guest interactions [17,18].

#### 2.1.2. Metal-Organic Gels (MOGs)

Metal-organic gels are a class of functional soft materials with a three-dimensional, percolating network and solid-like rheological behaviour. The framework of MOGs is constructed from metal ions or metal-containing clusters that are bridged by multi-dentate organic ligands through metal-ligand coordination. Additional non-covalent interactions, such as hydrogen bonding, π–π stacking, hydrophobic interactions and van der Waals forces, are often present as well [16,17,19,20,21].

**Figure 3:**
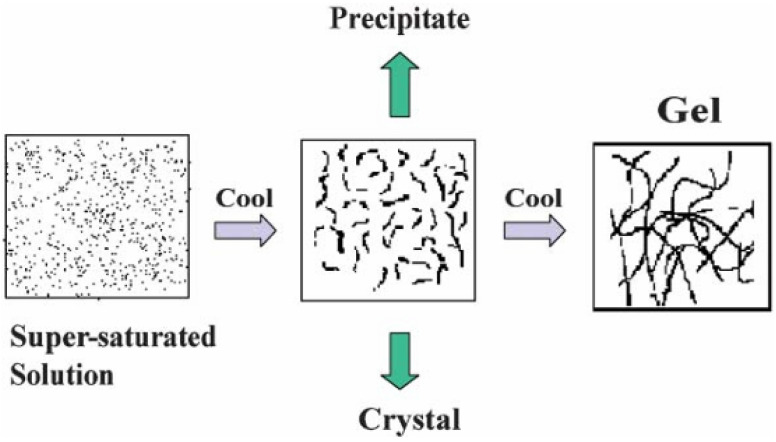
Schematic diagram of organometal gel aggregation process [11].

Structurally, MOGs can be considered MOF- or coordination-polymer-like networks in a gel state. They typically exhibit hierarchical micro-/mesoporous architectures, a high specific surface area and accessible active sites. The porosity varies by synthesis route—not every MOG is highly porous [11,17,20]. Their formation mechanisms include the mismatched or arrested growth of coordination polymers/MOF particles, the sol–gel conversion of metal-organic networks, and the supramolecular self-assembly of discrete metal complexes into a space-spanning network [17,20]. A schematic representation of the aggregation and gelation process in metal-organic gels is shown in Figure 3. Due to their combination of inorganic nodes (metal ions or metal-oxo clusters) and organic linkers, MOGs can be tuned in composition, structure, and mechanical properties. They also exhibit a variety of functional behaviours, including adsorption, catalysis, ion/electron transport, and stimuli-responsiveness [16,19,20,21]. Consequently, they have been widely explored as precursors to porous carbons and metal oxides, adsorbents, heterogeneous catalysts, and electrochemical materials, including electrocatalysts, supercapacitor/battery electrodes and electrolytes, and platforms for drug delivery and other pharmaceutical applications. In the broader context of soft matter, MOGs are typically classified as metal–organic or coordination-polymer gels. These gels are the gel-state analogues of metal–organic frameworks and coordination networks. They are distinguished from conventional organogels or polymer hydrogels by the dominant structural role of metal-ligand coordination within the gel scaffold [16,17,19,20,21].

#### 2.1.3. Metal–Phenolic Gels (MPGs)

Metal–phenolic gels are a class of supramolecular, coordination-based soft materials formed through the assembly of metal ions (or metal–oxo clusters) with polyphenolic ligands—typically catechol- or gallol-containing molecules, such as tannic acid—via metal-ligand coordination bonding. This process is often accompanied by additional non-covalent interactions, including hydrogen bonding, π–π stacking, and hydrophobic forces. These interactions drive the formation of an extended, three-dimensional, percolating network, giving the material solid-like rheological properties despite its high water content. This places MPGs within the broader category of coordination-driven hydrogels and metallogels [22,23,24,25]. MPGs are amorphous metal–organic networks rather than crystalline frameworks, and the resulting disordered coordination matrix allows the incorporation of diverse metal ions (e.g., Fe^3+^, Al^3+^, Ti^4+^, and Zn^2+^), producing materials with tunable mechanical, chemical, and dynamic behaviour.

**Figure 4:**
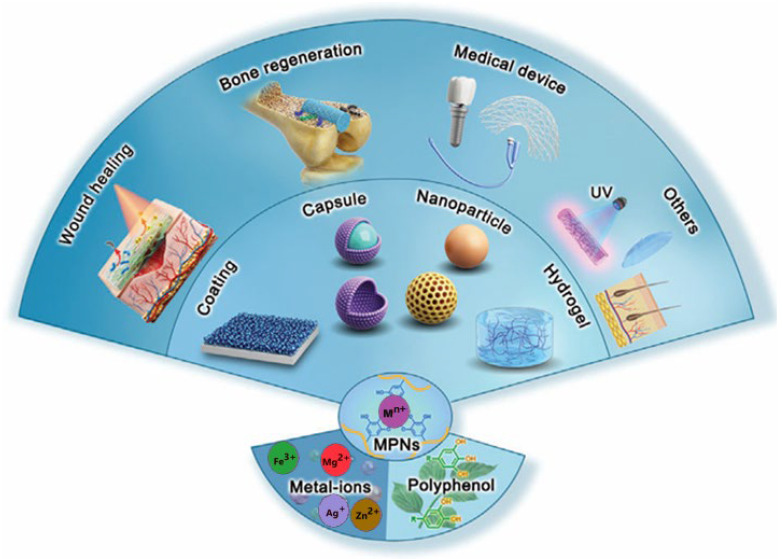
Fabrication Pathways and Structural Diversity of Metal–Phenolic Networks and Their Antimicrobial Applications Across Biomedical Disciplines [25].

Their rapid, aqueous, and mild self-assembly, enabled by the deprotonation and chelation of phenolic groups, supports the formation of gels, films, coatings, capsules, or particles, depending on the stoichiometry, pH, and metal-ligand ratios [22,23,24]. The coordination bonds and supramolecular interactions within MPGs are reversible and environmentally responsive, meaning MPG materials often exhibit pH-dependent behaviour, redox sensitivity, and dynamic disassembly/reassembly. This makes them attractive for use in drug delivery, biomedical coatings, wound healing, catalysis, sensing, and interface engineering. Integrating inorganic metal nodes with multifunctional phenolic ligands imparts adhesive, antioxidant, antimicrobial, and catalytic properties, expanding their applicability to chemistry, biomedicine, and materials science [23,24,25]. The diversity of fabrication pathways, structural motifs, and representative antimicrobial applications of metal–phenolic networks is illustrated in Figure 4.

#### 2.1.4. Metal Nanoparticle-Based Gels (MNPGs)

Metal nanoparticle-based gels are a class of soft materials in which a self-supporting, three-dimensional network forms through the assembly, aggregation or cross-linking of metal nanoparticles. This structure exhibits solid-like rheological behaviour despite a high solvent content. In these systems, the metal nanoparticles—typically composed of noble metals (e.g., Au, Ag, Pt), transition metals or metal oxides—function as the primary structural building units and gelation arises from interparticle interactions including ligand-mediated bridging, supramolecular binding, surface-surface fusion or coordination-driven association of the nanoparticles [26,27,28]. As the nanoscale metallic domains retain their catalytic, optical, redox, plasmonic, antimicrobial or conductive properties, MNPGs combine the functional properties of metal nanoparticles with the mechanical characteristics of soft gels. This hybrid nature enables tunable viscoelasticity, responsiveness to chemical or physical stimuli, enhanced catalytic performance, and improved electronic or thermal transport within the gel matrix [26,27,28,29]. MNPGs can be formed either by the direct self-assembly of metal nanoparticles into a gel network or by embedding the nanoparticles within a polymeric or supramolecular gel matrix. In the latter case, the nanoparticles play a critical role in the mechanical integrity or functional behaviour of the gel. Due to their structural versatility and functional richness, MNPGs have been investigated for use in heterogeneous catalysis, sensing and detection, soft electronics, photothermal systems, tissue engineering and wound healing, in situations where the interaction between the nanoparticulate phase and the gel environment is beneficial [26,27,28,29,30].

The direct use of metal nanoparticles in biomedical applications is limited by factors such as aggregation, rapid clearance, and potential toxicity. However, incorporating them into hydrogels stabilises the nanoparticles, reduces aggregation, and prolongs local retention while providing a hydrated, tissue-like environment [26,27]. In such hybrid materials, the hydrogel network serves as a physical entrapping agent for the nanoparticles and also contributes to rheological properties, controlled release, and responsiveness to external stimuli (e.g., pH, temperature, and light) [26,29,31].

**Figure 5:**
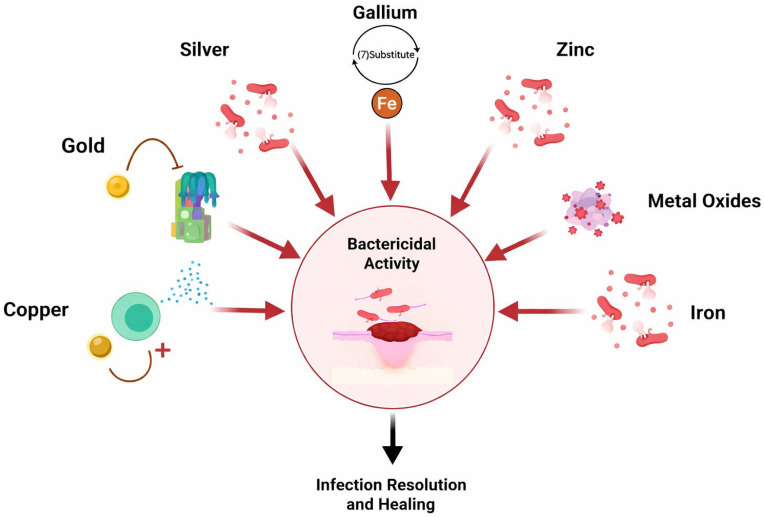
Schematic representation of bactericidal mechanisms of metal-based nanoparticles and their contribution to infection and wound healing [28].

A systematic review of metal nanoparticle-embedded hydrogels for in vivo wound healing shows that hydrogels containing silver, zinc, gold, iron, or gallium nanoparticles can exhibit strong antimicrobial activity, reduced inflammation, enhanced angiogenesis, and accelerated wound closure in animal models. For instance, iron- and gallium-containing hydrogels have been shown to eradicate *Staphylococcus aureus* and *Escherichia coli* infections, while also promoting collagen deposition and tissue regeneration in full-thickness wound models [27]. These results highlight that, in nanoparticle-forming metallogels, the metallic component contributes directly to antimicrobial and pro-regenerative effects, while the hydrogel matrix provides local retention, biocompatibility, and tunable mechanical support. Figure 5 schematically summarises the principal bactericidal mechanisms of metal-based nanoparticles and their role in infection control and wound healing. In addition to wound healing, noble metal nanoparticles combined with hydrogel matrices have been widely used for sensing and catalysis. A review of nanoparticle–hydrogel-based sensors discusses how incorporating noble metal nanoparticles (e.g., Au, Ag and Pt) into hydrogels creates composite materials with enhanced catalytic and optical properties. These materials can be engineered into sensor platforms that respond to stimuli [15,29,31]. The hydrogel’s porous network stabilises the nanoparticles, facilitates mass transport and can be chemically tailored for selective analyte recognition. Experimental work on noble metal nanoparticle-based hydrogel composites for the colourimetric detection of heavy metals further illustrates that the in situ formation of Au, Ag and Pt nanoparticles within polymer networks produces robust materials that exhibit clear, analyte-dependent colour changes, which can be structurally characterised using electron microscopy, XRD and FTIR [32,33].

#### 2.1.5. Polymer-Based Metallogels (PMGs)

Polymer-based metallogels are soft materials comprising a three-dimensional, solvent-swollen network formed by coordination interactions between metal ions and ligand functionalities on polymer chains. This produces a solid-like, percolating structure [15]. The polymer backbone acts as the primary structural scaffold, and the metal-ligand coordination junctions act as either reversible or permanent cross-links. These cross-links govern the material’s mechanical strength, viscoelasticity, and responsiveness to environmental stimuli, such as pH, redox conditions, or competing ligands. Incorporating metal coordination motifs into polymer matrices endows the resulting materials with functional properties, including self-healing, catalytic activity, ion/electron transport, and stimuli-responsive behaviour. These properties exceed the capabilities of purely organic hydrogels. Due to this hybrid synergy between polymer flexibility and metal-derived functionality, PMGs have found applications in drug delivery, sensing, catalysis, soft electronics, and biomaterials engineering [14,15,18].

#### 2.1.6. Low-Molecular-Weight Metallogels (LMW-MGs)

Low-molecular-weight metallogels (LMW-MGs) are supramolecular soft materials in which a three-dimensional network that immobilises the solvent forms through the self-assembly of low-molecular-weight gelator molecules (typically <1500 Da) induced by metal ions. The discrete structures of these molecules allow highly controlled, coordination-driven aggregation [17]. Gelation occurs when metal ions bind to ligand sites on small gelators, such as pyridyl derivatives, salicylidene/Schiff-base molecules, hydrazone-bearing aromatic compounds, or short peptide-based gelators, creating directional coordination bonds that nucleate higher-order structures. These coordination interactions cooperate with secondary non-covalent forces, such as hydrogen bonding, π–π stacking, and hydrophobic contacts, to yield fibrillar or particulate supramolecular networks. This has been demonstrated in systems formed by small gelators coordinated by Cu^2+^, Zn^2+^, Fe^3+^, Ni^2+^, or Co^2+^ ions [16,17,18,34]. As these gelators are low-molecular-weight and non-polymeric, the coordination event itself acts as the dominant structural trigger. This leads to rapid assembly, reversible gel–sol transitions and strong sensitivity to environmental stimuli, such as pH, redox conditions or ligand competition [16,18]. Incorporating metal centres into these minimal frameworks can impart catalytic, optical, magnetic, or redox activities, enabling LMW-MGs to function as chemical sensors, catalytic media, templates for nanostructure formation, controlled-release matrices, and responsive smart materials [16,17,18,34] (Table 1).

### 2.2. Based on Type of Metal Interaction

Classifying metallogels based on the type of metal interaction reflects the fact that the nature of the metal-based structural motif fundamentally determines the assembly pathway, network architecture, and emergent properties of the resulting gel. Different modes of metal interaction—ranging from classical coordination bonding to nanoparticle formation to redox-regulated assembly—give rise to distinct subclasses of metallogels, each characterised by unique chemical principles and functional behaviours.

#### 2.2.1. Coordination-Driven

Coordination-driven metallogels are characterised by metal-ligand coordination playing a central role in forming a continuous three-dimensional network. This type of metallogel is well documented in the literature. It has been documented that metallogelators can originate from discrete metal complexes and coordination polymers, and that these coordination-based species can assemble into supramolecular structures that form gel phases [17]. This establishes coordination bonding as a fundamental driving force in metallogel formation. Metallogels are explicitly classified as materials derived from discrete coordination complexes, organometallic complexes and coordination polymers, reflecting the fact that coordination-driven systems constitute a distinct and recognised subgroup [35]. Further analysis of coordination polymer gels shows that extended metal-ligand linkages can generate polymeric coordination networks that transition into gel states [36]. Additionally, the literature describes metallogels composed of transition-metal complexes, coordination polymers, and MOF-like structures, demonstrating that coordination-derived architectures at multiple structural levels can induce gelation [37]. Coordination-driven metallogels also exhibit properties that are characteristic of their coordination origin. Reviews of metallogel systems discuss the relevance of coordination complexes to material behaviour and applications, including environmental and biological uses [5]. Reported examples of stimuli-responsive metallogels demonstrate that dynamic coordination sites enable reversible structural or mechanical adjustments in response to environmental stimuli [16]. Surveys of metal-ion hydrogels further emphasise that gelation can be governed by coordination interactions between metal ions and functional ligands, influencing mechanical and functional characteristics [14].

#### 2.2.2. Discrete Metal-Complex-Based Metallogels

Although metallogels based on metal-ligand complexes and coordination-driven metallogels both rely on metal-ligand interactions, there are differences in their structural origin, degree of network extension, and gelation pathway. Metallogels based on metal-ligand complexes originate from discrete coordination complexes that self-assemble into gel networks through a combination of coordination bonding and auxiliary supramolecular forces [17,35]. These networks are not formed as continuous coordination frameworks, but rather arise from the supramolecular association of molecular coordination units. This often yields materials with notable tunability and responsiveness due to the dynamic nature of the complexes. The chemistry of these complexes, which exhibits soft-matter characteristics, grants them a unique position within the broader metallogel landscape [16]. Metal-ligand complex-based gels differ in that they rely on molecular complexes as building blocks, whereas coordination-driven gels depend on extended coordination architectures. While both categories reflect coordination chemistry, they differ in terms of the scale of assembly, the type of network propagation, and the resulting mechanical and functional characteristics.

#### 2.2.3. Lonic Cross-Linking Metallogels

Ionic cross-linking metallogels are gel systems in which multivalent metal cations act as physical cross-linkers, electrostatically bridging the charged functional groups of polymeric or supramolecular chains. Unlike coordination-driven metallogels, where metal-ligand coordination bonds dominate network formation, ionic cross-linking relies primarily on ion-mediated, non-covalent ionic interactions between metal ions and anionic groups, such as carboxylates, sulphates or phosphates [29,38]. These interactions lead to the formation of reversible junction zones that stabilise a three-dimensional, solvent-swollen network. In this context, ionic cross-linking hydrogels are classified as metallogels due to the essential structural role of metal ions in network formation, despite the predominance of electrostatic rather than directional coordination interactions.

This gelation mechanism is most commonly observed in hydrogels based on anionic biopolymers, including alginate, pectin, carrageenan, hyaluronic acid, and carboxymethyl cellulose, which rapidly gel upon exposure to divalent or trivalent metal ions such as Ca^2+^, Mg^2+^, Zn^2+^, Cu^2+^, Fe^3+^, or Al^3+^ [29,38,39]. A classical example is the Ca^2+^–alginate system, commonly described by the “egg-box” model, in which calcium ions are cooperatively bound within cavities formed by aligned guluronate residues, generating ion-mediated cross-linking domains along adjacent polymer chains [40,41,42]. Similar ion-bridging mechanisms have been reported for other polysaccharide systems and metal ions, with the strength and density of cross-links depending on ion valence, ionic radius, and polymer composition [14,38,43].

Ionic cross-linking metallogels are typically formed under mild, aqueous conditions and exhibit rapid gelation kinetics, high water content, and dynamic network behaviour. Because the ionic cross-links are reversible and labile, these gels often display shear-thinning, self-healing, and ion-exchange properties, but may also be susceptible to mechanical weakening or structural rearrangement in the presence of competing ions or chelating agents. Despite these limitations, ionic cross-linking metallogels are widely used in biomedical, food, and environmental applications due to their injectability, biocompatibility, and responsiveness. Their classification as metallogels reflects the essential structural role of metal ions in network formation, even though the dominant interactions are electrostatic rather than directional coordination bonds.

#### 2.2.4. Nanoparticle-Forming Metallogels

As MNPGs were described in general terms in Section 2.1.4. It is also notable that recent reviews on metal nanoparticle hybrid hydrogels describe how metallic nanoparticles, which exhibit rich optical, electronic, and catalytic behaviour, can be immobilised and organised within hydrogels to form composite systems that are mechanically robust, environmentally responsive, and multifunctional [26]. Nanoparticle-forming metallogels can be classified according to the dominant interaction mechanism responsible for network formation. One common approach is to use ligand-bridged nanoparticle networks, where dynamic metal-ligand coordination results in the formation of metal-ligand nanoparticles in situ. These nanoparticles function as multifunctional crosslinking nodes. Bisphosphonate–metal coordination is a good example of this, with self-assembled ligand–metal nanoparticles binding multiple polymer chains and inducing rapid gelation. The resulting nanocomposite hydrogels exhibit metal-dependent nanoparticle size, connectivity and mechanical response, thus demonstrating that coordination chemistry directly governs network formation at the nanoscale [44]. A second mechanism is surface-coordination-driven nanoparticle association, in which ligands tethered to nanoparticle surfaces interact cooperatively with metal ions or neighbouring particles. Terpyridine-functionalised gold nanoparticles demonstrate how weak individual surface interactions can collectively interlink nanoparticles when surface coverage is sufficiently high, resulting in aggregation and network formation. The reversibility and extent of this assembly are dictated by the coordination environment and metal-ligand complexation conditions [45]. Nanoparticle gelation may also be driven by non-specific physical interactions, such as van der Waals forces and depletion attractions. Polymer-induced depletion forces have been shown to induce the gelation of plasmonic metal oxide nanocrystals into space-spanning, arrested networks, whilst preserving the identities of the individual particles. Small-angle X-ray scattering and electron microscopy confirm that gelation in these systems arises from a balance of short-range attractions and long-range electrostatic repulsion rather than chemical fusion of nanoparticles [46]. Finally, the formation of nanoparticles within a pre-existing gel network represents an alternative pathway, whereby the gel phase itself stabilises the nanoparticles and constrains their spatial organisation. Supramolecular organogels and polysaccharide gels have been shown to act as reducing agents, templates and supports for gold nanoparticle formation simultaneously, yielding hybrid materials in which nanoparticle diffusion is suppressed by the fibrous gel network [47,48]. Overall, these examples demonstrate that the formation of metallogels capable of forming nanoparticles is best understood and classified by the interaction mechanisms leading to gelation.

#### 2.2.5. Redox-Active Systems

Redox-active metallogel systems are metallogels in which the oxidation state of the metal centre or a redox-active ligand is crucial for gel formation, gel–sol transitions or functional responses. These materials can be induced to undergo reversible changes in structure, assembly mode, or optical and electronic properties by external redox stimuli, such as chemical oxidants/reductants or applied electrochemical potentials. This makes redox-active metallogels a key subclass of stimuli-responsive soft materials. These systems are discussed in detail as having potential applications in drug delivery, sensing, and energy storage because redox switching can control diffusion, loading/release behaviour, and electronic and optical properties [16]. The ferrocenyl group is highlighted as a prototypical redox centre that readily oxidises to a cationic state and has been widely used to construct redox-responsive metallogels. A ferrocene-based organometallic gel formed from a ferrocene–dipeptide gelator in a mixture of solvents exhibits reversible gel–sol transitions when the ferrocene unit is oxidised or reduced, demonstrating that the redox state of ferrocene directly controls the integrity of the supramolecular network [49]. In another system, a low-molecular-weight ferrocene-based metallogel exhibits multistimuli responsiveness, in which the ferrocene redox state, along with host–guest interactions, governs the network’s stability and morphology [16,49]. Redox-active organic radical gelators provide an alternative approach: nitronyl nitroxide-based small molecules can form redox-active supramolecular fibres and physical gels. The redox behaviour of the radical is confirmed by cyclic voltammetry, demonstrating that purely organic redox centres can also be exploited to create redox-responsive gel networks [47]. Redox-active metallogels centred on transition metals are equally well documented. For example, a copper(II) quinolinol-based metallogel has been reported to undergo redox-responsive gel–sol–gel cycling. Reduction causes the gel to collapse into a sol, and subsequent oxidation restores the gel. These transitions are accompanied by changes in supramolecular chirality and morphology. The gel state also exhibits enantioselective recognition of aromatic amino acids [8]. Another archetypal redox-switchable system is a copper(I) metallogel formed from thiourea and copper chloride: the metallogel is generated via the in situ reduction of Cu(II) to Cu(I), followed by Cu(I)-thiourea coordination. The resulting gel exhibits reversible gel–sol transitions driven by redox changes in the Cu(I) centres, while maintaining its entangled network morphology. This same system functions as a highly selective naked-eye fluorescent sensor for picric acid, demonstrating how redox-active metal centres can couple phase behaviour with analyte detection [50]. Beyond sensing applications, redox-active supramolecular gels have been used in energy storage and soft electronic devices. For example, a benzoylpyridinium-substituted supramolecular gelator with n-type redox properties forms a robust organogel, in which the reversible redox reactions of the benzoylpyridinium units enable charge/discharge processes around 1.05 V versus Fc/Fc^+^. This gel has been successfully used as part of a flexible, transparent, gel-based, rechargeable device [51]. This example highlights that redox-active gels can function as electrode-active, charge-storing soft materials, linking supramolecular gel structures with electrochemical energy storage (Table 2).

### 2.3. Based on Function

In addition to their structural and mechanistic differences, metallogels can also be categorised based on their primary functional characteristics. Due to the unique physicochemical properties imparted by metal ions, metal complexes, and metallic domains, metallogels often act as active materials rather than passive matrices. Their metal-derived features, such as catalytic activity, electronic conductivity, optical emission, antimicrobial behaviour, redox activity, and therapeutic performance, allow them to function as versatile soft materials in chemical, technological, and biomedical contexts.

#### 2.3.1. Catalytic Gels

**Figure 6:**
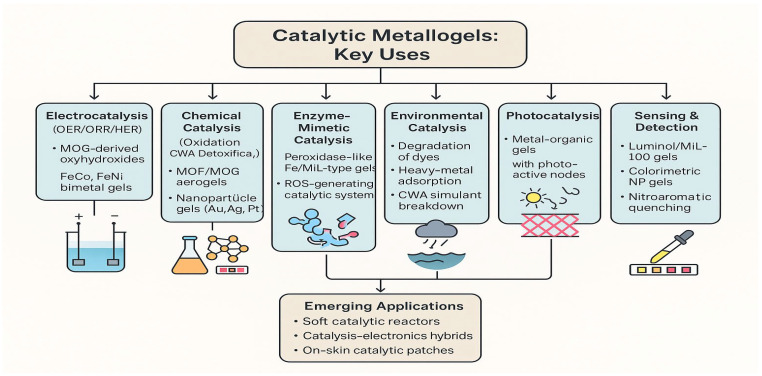
Catalytic Application Landscape of Metallogels.

Catalytic metallogels are defined as gel systems in which metal-containing sites, which act as accessible catalytic centres for chemical or electrochemical transformations, are distributed throughout the gel network. A notable example is MOGs, which combine the high surface area, hierarchical porosity and numerous metal active sites of MOFs with the processability of a gel-state material. These MOGs have been extensively investigated for the adsorption and catalytic detoxification of highly reactive and persistent chemical warfare agents and their simulants, due to their large specific surface area, interconnected pore structure, and catalytic metal nodes [53,54]. Figure 6 provides an overview of the major catalytic application areas of metallogels.

The catalytic role of metal-organic gels is particularly evident in oxygen electrocatalysis. A detailed analysis of metal-organic frameworks and gels for oxygen evolution and reduction has shown that MOGs can act as active electrocatalysts and as precursors to highly active oxyhydroxide phases. Design strategies focus on maximising active-site density, controlling porosity and tuning metal composition [55]. Phytic acid-based FeCo bimetallic metal-organic gels exemplify this concept: gels synthesised from phytic acid and Fe^3+^/Co^2+^ ions, then converted into aerogels, demonstrate outstanding performance in the oxygen evolution reaction, characterised by low overpotential and a small Tafel slope in alkaline environments [56]. More recently, the electrochemical reconstruction of Fe–Ni metal-organic gels into NiO(OH)/FeO(OH) heterostructures has been shown to generate highly active oxygen-evolution precatalysts, in which the initial MOG structure serves as a template for forming a catalytically optimised heterointerface [57]. Catalytic metallogels are also relevant in oxidative chemiluminescence systems. MIL-100 gels, constructed from Fe^3+^ and trimesic acid, have been demonstrated to possess oxidase-like catalytic activity. They efficiently catalyse luminol chemiluminescence by accelerating the generation of reactive oxygen species, enabling the highly sensitive detection of uric acid in biological samples [58]. These examples collectively demonstrate that catalytic metallogels utilise gel-confined metal centres, whether in MOF-like nodes, multinuclear sites or nanoparticulate domains, to facilitate various transformations, including electrocatalytic water splitting, the detoxification of hazardous substances and enzyme-mimicking oxidation reactions.

#### 2.3.2. Conductive/Metallo-Electronic Gels

Conductive, or metallo-electronic, metallogels are systems in which metal-containing networks or metal-rich domains confer semiconducting or electronically conductive behaviour. This enables them to be integrated into electronic and optoelectronic devices. Several supramolecular metallogels comprising divalent metal ions and low-molecular-weight dicarboxylic or amino-dicarboxylic acids have demonstrated clear semiconducting properties when incorporated into metal–semiconductor junctions. For example, a nickel(II) metallogel formed from 5-aminonaphthalene-1,8-bis(dicarboxylate) in N, N-dimethylformamide exhibits viscoelastic gel behaviour, a flake-like hierarchical microstructure, and an optical band gap in the semiconducting range. Thin films of this gel in indium tin oxide /metallogel/gold (Au) junctions behave as Schottky diodes with non-linear current–voltage characteristics [59]. A series of Zn(II)-based metallogels derived from the same or related low-molecular-weight gelators exhibit similar semiconducting behaviour and have been used to fabricate efficient Schottky barrier diodes with measurable electrical conductivities and well-defined rectification [60,61,62]. Copper-containing metallogels are another example of metallo-electronic behaviour. For instance, a supramolecular Cu(II) metallogel prepared from L-(+)-tartaric acid has been demonstrated to exhibit mechanical robustness, self-healing properties, and electrical activity. Devices constructed as metal–semiconductor junctions using this gel have been shown to exhibit Schottky barrier diode characteristics, confirming the existence of a continuous semiconducting pathway within the metallogel network [63]. Similarly, a related Cu(II) metallogel based on succinic acid-derived gelators exhibited semiconducting optical properties, and its integration into a Schottky diode configuration as a thin film substantiated its potential for microelectronic applications [64]. Conductive metallogels are not restricted to simple dicarboxylate systems. For example, a Zr-cluster-based metallogel has been reported to be thermostable, self-healing and electrically conductive. It has also been found to exhibit chromogenic and multi-stimuli-responsive properties in response to light, aliphatic amines, electrical input and metal exposure [65]. Furthermore, antibacterial scaffolds consisting of Ni(II)- and Zn(II)-metallogels have been developed that can function simultaneously as active semiconducting elements in light-responsive junction-type diodes, demonstrating the convergence of electronic and biomedical functions within a single material platform [66]. Collectively, these studies demonstrate that metallogels can act as soft semiconductors with tunable band gaps and device-relevant charge-transport properties, making them suitable for flexible electronics, sensors, and hybrid bioelectronic systems.

#### 2.3.3. Luminescent Metallogels

Luminescent metallogels are metallogels in which the photoluminescence of the metal centres, ligands or metal-ligand ensembles is preserved or enhanced in the gel state. This enables applications in optical sensing, imaging and anti-counterfeiting. Several classes of luminescent metal-organic gels have been reported, including those based on aggregation-induced emission (AIE) chromophores, lanthanide ions and metal nanoclusters. One example is metal-organic gels formed from the AIE-active tetraphenylethylene derivative tetrakis(4-carboxyphenyl)ethylene (H_4_TCPE) and trivalent metal ions such as Al^3+^, Cr^3+^, Fe^3+^, Ga^3+^, and In^3+^. These gels, especially the Al–TCPE gel, combine hierarchical micro- and mesoporosity with strong AIE photoluminescence. The corresponding aerogels consist of MOF-like nanocomponents and have been shown to act as luminescent sensors for nitroaromatic explosives, such as picric acid [67]. ZrBDC gels derived from UiO-66 that are trace-doped (down to 0.01 mol%) with H_4_TCPE exhibit remarkably enhanced luminescence. These gels retain the porosity of the parent gel while achieving high quantum yields and high sensitivity and selectivity towards nitroaromatic analytes [68]. Zr-based MOF gels prepared from tetracarboxylate linkers, such as pyrene- or tetraphenylethylene derivatives, also exhibit strong luminescence. These gels have been used as sensors for volatile organic compounds and nitroaromatics in both solution and vapour phases, due to their rapid quenching responses and high surface areas [53]. Transition-metal-based luminescent metallogels offer additional functionality. For example, a zinc(II)-terpyridine metal–organic gel exhibits visible luminescence that is modulated by anion binding at the zinc centres. This enables visual recognition of anions through changes in emission [69]. Luminescent hydrogels based on silver nanoclusters stabilised by malic acid deliver intense fluorescence and have been incorporated into composite films capable of highly sensitive Fe^3+^ detection by exploiting the quenching of nanocluster emission upon metal binding [70]. Lanthanide-containing metallogels and hydrogels are another important class of luminescent systems. Self-assembled Eu^3+^/Tb^3+^ supramolecular gels exhibit high luminescence and self-healing properties, with colour tunability achieved by varying the Eu:Tb ratio. Rheological and imaging studies demonstrate that lanthanide ions play a crucial role in supramolecular polymerisation and the resulting gel properties. Similarly, cross-linked lanthanide hydrogels derived from a bis(triazolyl)picolinamide ligand display strong Tb^3+^- and Eu^3+^-centred emissions in the gel state [62,63,68]. A heat-set Tb-based metallogel prepared from a triazine-tris(isophthalate) ligand retains luminescence over a broad temperature range and in response to mechanical and aqueous stimuli, demonstrating its potential for use in stable optical and anti-counterfeiting materials [71]. More recently, lanthanide-based metallogels have been engineered for tunable luminescence and the nanomolar detection of nerve agent simulants, as well as for anticounterfeiting applications. This demonstrates the integration of sensing and security functionalities within luminescent gel frameworks [72]. Overall, luminescent metallogels are a versatile class, as the soft nature of the gel and the optical properties of the metal-containing components synergistically enable responsive, high-contrast photonic functions.

#### 2.3.4. Antimicrobial & Biomedical Metallogels

Antimicrobial and biomedical metallogels are systems in which the metal component and the gel network work together to perform therapeutic, antimicrobial, or tissue-regenerative functions. A recent comprehensive review of supramolecular metallogels for biomedical applications emphasises their potential in targeted drug delivery, antimicrobial therapy, wound healing, and imaging. This versatility is attributed to dynamic metal-ligand architectures that provide tunable mechanical properties, controlled release, and the ability to generate reactive oxygen species or incorporate therapeutic payloads [12].

Metal–phenolic metallogels demonstrate how coordination chemistry can be utilised in wound dressings. A tannic acid–Ti(IV) metallogel has been developed as a naturally derived platform that can incorporate antimicrobial metal ions, such as Fe^3+^, Cu^2+^, Zn^2+^, Co^2+^, and Ni^2+^, through co-gelation. These gels demonstrate pH- and H_2_O_2_-dependent release of metal ions, which matches the acidic and oxidative microenvironment of infected wounds. They also display strong antimicrobial activity against Gram-negative *Escherichia coli* and Gram-positive methicillin-resistant *Staphylococcus aureus* and *Staphylococcus epidermidis*. In vivo studies in infected wound models demonstrate accelerated healing and reduced bacterial load compared to control gels and gauze, confirming their potential as intelligent wound dressings [73]. Hybrid systems that combine hydrogels with metallic nanoparticles also naturally fall within this functional class. Metallic nanocomposite hydrogels, which contain embedded silver, zinc oxide, iron, or other nanoparticles within polymer networks, have been extensively reviewed for use as biocompatible, biodegradable, and antimicrobial wound dressings that can promote faster wound closure, control bacterial growth, and replace conventional dressings [74].

A dedicated analysis of hydrogel scaffolds embedded with metallic nanoparticles shows that these systems can provide a moist, protective environment, with the nanoparticles acting as local antimicrobial agents to improve healing outcomes and control infection in vivo [67]. More broadly, metal-organic frameworks, including gel-like morphologies, have been reported as promising platforms for infectious wound healing, where their metal nodes and porous structures contribute to antibacterial efficacy, reactive oxygen species generation, and the controlled release of therapeutic agents [75]. There is a growing overlap between antimicrobial and electronic functions. Ni(II)- and Zn(II)-based metallogels, for example, have been demonstrated to form antibacterial scaffolds that simultaneously serve as semiconducting elements in light-responsive junction-type diodes, suggesting future applications in implantable or on-skin bioelectronic devices that integrate antimicrobial activity with sensing or signal transduction [66]. Altogether, antimicrobial and biomedical metallogels are characterised by their ability to couple gel mechanics, metal-based reactivity, and biological interactions, positioning them as multifunctional soft materials for infection control, regenerative medicine, and theranostics.

Metallogels offer emerging opportunities as multifunctional platforms for infection control, integrating antimicrobial activity with dynamic responsiveness. Hydrogels coordinated by metal ions, incorporating Ag^+^, Cu^2+^, Zn^2+^, Fe^3+^ or Mn^2+^, have demonstrated efficient bacterial killing in infected wound models, while also modulating inflammation and promoting angiogenesis [69,76]. Furthermore, metal–phenolic coordinated hydrogels enable antibacterial action in response to stimuli, such as acidic pH and endogenous H_2_O_2_, which are present in infected tissues and trigger metal release or catalytic chemodynamic reactions. This results in the effective eradication of biofilms in vivo [74]. Recent designs also integrate photothermal and chemodynamic therapies within metallogel networks, achieving synergistic disruption of biofilms and accelerated wound closure whilst enabling real-time monitoring of wound status [77]. Beyond infection control, metallogels are being increasingly explored for regenerative medicine applications due to their ability to regulate the immune microenvironment and support tissue repair. Metal ion–crosslinked hydrogels that release Mg^2+^ or Zn^2+^ have been shown to promote the polarisation of macrophages towards a pro-regenerative phenotype and to enhance the differentiation of stem cells, thereby improving cartilage regeneration and wound healing in animal models [78]. Reviews of metal ion–based hydrogels further highlight their capacity to mimic extracellular matrix functions, provide dynamic mechanical support and deliver bioactive cues for tissue engineering applications [79]. Metallogels and metal-coordinated soft nanomaterials present significant opportunities in theranostics, combining therapeutic and diagnostic functions within a single platform. Metal–phenolic networks and metal-coordinated supramolecular assemblies have been widely demonstrated to carry imaging agents and therapeutic modalities, including photothermal, chemodynamic and drug delivery systems, particularly in cancer models [80,81]. Similarly, theranostic nanogels that incorporate metal-based contrast agents can enable the simultaneous delivery of treatment and imaging, facilitating real-time monitoring of the therapeutic response and disease progression [82]. Taken together, these studies demonstrate the potential for metallogels to evolve from passive biomaterials into intelligent, multifunctional systems for precision medicine.

Despite their undoubted advantages, there are also many potential risks associated with metallogels, such as metal leaching, stability in physiological environments, and immune responses. Metal leaching is a key factor that influences the antimicrobial efficacy and biosafety of biomedical metallogels. Experimental studies show that the release of metals from metallogels is governed by the strength of metal-ligand coordination and environmental conditions rather than uncontrolled degradation. In metallogels containing phenolic compounds such as tannic acid, the sustained release of antimicrobial metal ions under physiological conditions (PBS, pH 7.4) has been quantified using inductively coupled plasma mass spectrometry (ICP-MS), with distinct release profiles observed for different metal species [74]. Notably, pathological cues such as acidic pH and endogenous hydrogen peroxide, which are commonly present in infected or inflamed tissues, significantly accelerate metal ion release by inducing metallogel disassembly [74]. Embedding metal species within hydrogel matrices has been shown to reduce burst release and prolong local retention compared to free metal nanoparticles, thereby mitigating toxicity while preserving antibacterial activity [27]. Nevertheless, gradual metal leaching remains unavoidable due to nanoparticle oxidation and long-term exposure to physiological fluids. This highlights the need for careful control of metal dosage and release kinetics in metallogel design [27,81]. Metallogel stability is highly environment-dependent and must be carefully considered for biomedical applications. The stability of biomedical metallogels is governed by their coordination chemistry and the surrounding physiological milieu. Metal–phenolic hydrogels based on tannic acid and Ti^4+^ exhibit good structural stability under physiological saline conditions and body temperature, remaining localised and intact in vivo for extended periods with minimal metal redistribution [83]. However, pathological environments can compromise this stability, as reactive oxygen species such as hydrogen peroxide generated during inflammation oxidise phenolic ligands and trigger partial network disassembly [74]. The interaction of metallogels with the immune system is crucial to their biomedical performance. In vivo studies of metal-phenolic hydrogels based on Ti^4+^-tannic acid demonstrate good immunocompatibility, characterised by a mild and localised foreign body response following subcutaneous injection. This is evidenced by limited immune cell infiltration and low systemic metal accumulation over extended implantation periods [83]. Histological analyses revealed the formation of a thin fibrotic capsule and gradual immune adaptation comparable to that observed with conventional biocompatible hydrogels. Beyond passive compatibility, emerging metallogels actively modulate immune responses through the controlled release of metal ions. Dynamic hydrogels coordinated with bioactive metal ions, such as Zn^2+^ and Mg^2+^, have been shown to regulate macrophage polarisation, suppress pro-inflammatory cytokines (e.g., TNF-α and IL-6) and promote a pro-regenerative M2 phenotype in infected wounds and cartilage repair models [78,83]. These immunomodulatory effects, coupled with antimicrobial activity, highlight the potential of metallogels as active regulators of the inflammatory microenvironment in regenerative medicine, not just as inert scaffolds. Cytotoxicity is also an important parameter in the case of metallogels for biomedical and antimicrobial applications. Silver nanoparticles (AgNPs), which are widely used due to their broad-spectrum antimicrobial activity, have been shown to exert dose- and context-dependent cytotoxic effects in mammalian and regenerative systems. In vitro studies using human fibroblasts and glioblastoma cells have demonstrated that AgNPs induce metabolic inhibition, mitochondrial dysfunction, reactive oxygen species (ROS) generation and DNA damage in a concentration- and time-dependent manner. This leads to cell cycle arrest predominantly at the G2/M phase rather than extensive apoptosis [84]. Further evidence from transmission electron microscopy revealed the intracellular localisation of AgNPs within mitochondria and nuclei, suggesting direct interactions between nanoparticles and organelles as a cause of cytotoxicity and genotoxicity [84]. In vivo evidence from a zebrafish fin regeneration model showed that exposure to AgNPs at clinically relevant concentrations impaired wound healing in a stage-dependent manner, particularly during epithelialisation and early blastema formation [85]. This inhibition was associated with reduced cell proliferation and altered inflammatory responses, including enhanced neutrophil recruitment, rather than increased ROS production or silver ion toxicity. Taken together, these findings suggest that, although AgNPs are effective antimicrobial agents, excessive or uncontrolled exposure can disrupt cellular metabolism, DNA integrity and tissue regeneration. This emphasises the importance of controlling the dose and regulating the time of exposure in biomedical applications (Table 3).

## 3. Synthesis Pathways

Metallogels can be obtained via several distinct synthetic routes, which differ in the methods used to introduce the metal species and to establish the three-dimensional network. Reviews of metallogels and coordination polymer gels emphasise that metal ions can be incorporated as discrete coordination complexes, cross-linking nodes, or nanoparticles adhering to an organic network. They also highlight that the preparation strategy has a strong impact on the resulting structure and function [35,37]. Furthermore, recent studies on metal-ion-assisted supramolecular gelation and the real-time monitoring of metallogel formation emphasise the significance of self-assembly, coordination geometry, and kinetic control in determining gelation pathways and final architectures [86,87].

### 3.1. Coordination Chemistry Approaches

In coordination chemistry, metallogels form when metal ions are combined with multidentate ligands (typically low-molecular-weight gelators or polydentate organic linkers) under conditions conducive to the formation of extended coordination networks. A tutorial review of coordination polymer gels describes how metal ions and bridging organic ligands form solid-like networks analogous to those of metal-organic frameworks. In these systems, coordination between the metal and polydentate ligands is central to the gel network, while additional non-covalent interactions stabilise the fibrillar structures [37].

Similarly, a focus review on metallogels derived from coordination complexes, organometallic gelators and coordination polymers treats metal-ligand coordination as the primary design element for metallogelation and explicitly notes that metal ions can appear as either coordinated nodes in coordination polymers or metal nanoparticles adhered to organic networks [35].

More recently, a review of metal-ion-assisted supramolecular gelation discussed a broad range of gels formed with metal-ion assistance, including those based on cholesterol derivatives, amino acids, peptides, nucleic acid derivatives, oxalic acid, and tris-urea scaffolds [86]. This work demonstrates how the choice of metal ion (coordination number and lability) and ligand structure can enable precise control over gelation behaviour, mechanical properties and responsiveness. A study on metallogels formed from bidentate gel-forming ligands and various metal ions, published in the journal Soft Matter, further demonstrates how metal coordination geometry and metal identity influence the arrangement of fibres and correlation length in the gel network. This was observed using small-angle neutron scattering and real-time SAXS [87].

Primary experimental studies using small-angle neutron and X-ray scattering techniques have shown that the geometry of metal coordination directly determines fibre arrangement and correlation length in coordination-driven gels. Real-time small-angle neutron scattering (SANS) and small-angle X-ray scattering (SAXS) investigations of bis(pyridyl)-urea metallogels demonstrate that metal ions with distinct coordination preferences generate quantitatively different nanoscale networks. Linear or low-coordination geometries, as seen with Ag(I), reinforce one-dimensional alignment of supramolecular building blocks, producing well-defined fibrillar bundles with relatively large, temperature-stable correlation lengths. In contrast, metal ions capable of higher coordination numbers or adaptive geometries, such as Fe(III), Dy(III) and Ho(III), induce branching, fibre collapse or laminated assemblies. This leads to shorter correlation lengths and reduced spatial correlation between fibres, as extracted directly from correlation-length model fitting of SANS data [86]. Correlation lengths ranging from a few angstroms to several hundred angstroms have been reported solely as a function of metal identity and coordination mode, thus confirming the causal relationship between coordination geometry and network length scale. Complementary experimental evidence from metallo-supramolecular polymer networks further supports this principle. By systematically varying the preferred coordination geometry of phenanthroline–metal junctions, distinct network connectivities and relaxation behaviours were obtained. This demonstrates that coordination geometry controls the effective junction functionality and spatial organisation of the network, rather than merely bond strength [88]. Taken together, these primary scattering and rheological studies confirm that coordination geometry acts as a structural programming element that governs fibre arrangement, persistence and correlation length in metallogels.

Also the class of metal ion plays a critical role in coordination-driven metallogels, as differences in coordination geometry and directionality directly influence fibre arrangement and correlation length. Transition metals typically adopt well-defined, directional coordination geometries that promote anisotropic growth of supramolecular assemblies, leading to relatively ordered fibrillar networks with measurable and metal-dependent correlation lengths, as demonstrated by SANS and SAXS analyses of bis(pyridyl)-urea metallogels [86]. In contrast, lanthanide ions exhibit higher and more flexible coordination numbers with predominantly electrostatic bonding, which favours multivalent cross-linking and results in branched or disordered fibre networks with shorter correlation lengths and reduced long-range order [2,86]. Similar trends are observed in coordination polymer and metallo-supramolecular polymer gels, where reduced geometric constraint at the metal node leads to amorphous, weakly correlated network structures [88,89]. These findings establish coordination geometry and metal class as key parameters for controlling fibre organisation and length-scale hierarchy in metallogels.

Also metal coordination geometry plays a decisive mechanistic role in governing gelation kinetics, network architecture, and mechanical stability in metallogels. Directional and well-defined coordination environments promote anisotropic growth of supramolecular fibres, whereas higher coordination numbers and more isotropic binding motifs tend to generate branched or dynamically rearranging networks [90]. Early studies demonstrated that metal ions act not merely as passive crosslinkers but as structural regulators whose preferred coordination geometries dictate the packing and persistence of fibrillar assemblies [52]. In polymer-based and supramolecular metallogels, dynamic metal–ligand coordination allows for reversible crosslinking. The lifetime and geometry of the coordination bond directly influence stress relaxation and self-healing behaviour. Bio-inspired catechol–metal systems clearly illustrate this mechanism, with Fe^3+^, V^3+^ and Al^3+^ forming coordination complexes with different stoichiometries and geometries that result in significant variations in gel stiffness and relaxation dynamics under identical conditions [52]. At the supramolecular level, discrete metal complexes with square-planar or octahedral coordination geometries favour linear or ribbon-like aggregation, facilitating fibre entanglement and stable gel formation. In contrast, more labile coordination promotes adaptive, stimuli-responsive networks [91].

Within this framework, transition metals, characterised by tunable ligand field stabilisation and redox accessibility, enable precise control over gelation kinetics and reversibility through well-defined, directional coordination environments. In contrast, lanthanide ions, which typically exhibit higher coordination numbers and more isotropic electrostatic metal-ligand interactions, promote multivalent cross-linking and dynamically adaptive, yet less directionally ordered, gel networks [52,90,92]. Taken together, these studies establish coordination geometry as a key design parameter linking molecular-scale metal-ligand interactions to mesoscale network organisation and bulk gel properties.

### 3.2. In Situ Reduction to NP-Containing Metallogels

In situ reduction routes produce metallogels containing metal nanoparticles formed directly within a gel matrix. One example is the in situ synthesis of silver nanoparticles within a self-assembling, ultrashort peptide hydrogel. A tetrapeptide was designed to form a nanofibrillar hydrogel in an aqueous solution. Silver ions (Ag^+^) were then reduced photochemically in sunlight to generate Ag nanoparticles that were distributed uniformly inside the gel [91]. Spectroscopic and microscopic characterisation confirmed the presence of both the nanofibrillar peptide network and the silver nanoparticles. The resulting composite hydrogel exhibited enhanced antibacterial activity against methicillin-resistant *Staphylococcus aureus* and *Escherichia coli*, as well as promoting wound healing in cell assays, outperforming the peptide gel alone. Reviews of metal nanoparticle–hydrogel hybrids also emphasise that metal ions can be reduced in situ within polymeric or supramolecular gels to yield stable nanoparticle-gel composites that combine the mechanical and swelling properties of hydrogels with the catalytic, optical, or antimicrobial functions of metal nanoparticles [26,27,91,93].

These sources demonstrate that in situ reduction is a practical and widely employed approach for producing NP-containing metallogels, particularly for biomedical and catalytic applications, as the gel matrix can regulate the size, dispersion, and local environment of the nanoparticles.

### 3.3. Gelation Through Metal-Ligand Self-Assembly

Metal-ligand self-assembly is one of the most common ways of creating supramolecular metallogels. In this process, low-molecular-weight gelators containing ligand groups self-assemble into fibrous networks in the presence of metal ions, with metal–ligand coordination triggering and stabilising gel formation. Dastidar and co-workers review of metallogels describes many examples of discrete coordination complexes or coordination polymers based on small organic ligands forming extended networks capable of immobilising solvent. These systems are stabilised by coordination bonds, hydrogen bonding, π–π stacking, and van der Waals interactions [36].

**Figure 7:**
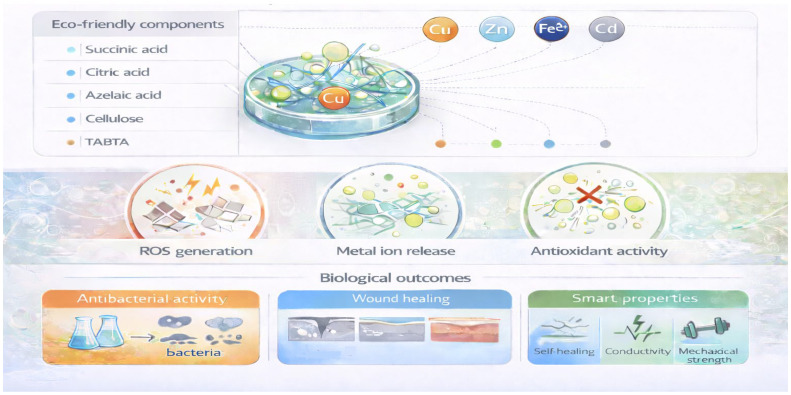
Schematic representation of multifunctional supramolecular metallogels developed for antibacterial and wound-healing applications (based on [12]).

A recent Pure and Applied Chemistry article explicitly focuses on metal-ion-assisted supramolecular gelation and presents cases in which adding metal ions induces or strengthens gelation in systems based on cholesterol derivatives, amino acids, peptides, nucleic acid derivatives, and tris-urea-based low-molecular-weight gelators [86]. Metal ions (Fe^2+^, Hg^2+^, Cd^2+^, Zn^2+^, and Cu^2+^) coordinate with organic gelators (e.g., GMP, succinic acid, citric acid, azelaic acid, cellulose, and TABTA), leading to the formation of hierarchical network structures with tunable mechanical properties and biologically relevant functionalitie [12]. Representative multifunctional supramolecular metallogels developed for antibacterial and wound-healing applications are illustrated in Figure 7.

This work confirms that metal ions can act as key triggers for gelation by forming coordination bonds with suitable ligand sites on the gelators. Metal-ligand self-assembly is also central to the functionality of metallogels, as demonstrated by a bis-terpyridyl low-molecular-weight gelator that forms a Cu^2+^-selective metallogel. In this system, coordination of copper(II) to terpyridyl units drives gelation, and the resulting gels exhibit multi-stimuli responsiveness (pH and temperature) and catalytic activity in click reactions [39].

Furthermore, a study in Scientific Reports has shown that gels formed through metal-ligand interactions can undergo reversible gel–sol transitions as the metal’s coordination state changes, demonstrating the dynamic nature of this self-assembly pathway [94].

These examples illustrate that metal-ligand self-assembly typically involves small ligands with well-defined coordination sites, in which metal addition leads to hierarchical self-assembly.

### 3.4. Diffusion-Mediated Formation

Diffusion-mediated formation exploits spatiotemporal control of gelation by gradually allowing either metal ions or ligands to diffuse into a solution or pre-gel medium containing the complementary component. While most metallogel studies rely on bulk mixing, research into supramolecular hydrogels has demonstrated that controlling the spatial distribution of gelation triggers can produce patterned or gradient structures. For instance, an article in Gels on the nanoscale spatial control of small-molecule hydrogelator self-assembly shows that self-assembly and gelation can be regulated by localising gelation triggers and using diffusion. This results in hydrogels with spatially controlled architectures [95]. Coordination polymer gels have been identified as dynamic systems in which self-assembly can be directed by external stimuli, such as concentration gradients and the slow addition of metal ions. These factors influence the final network morphology and properties [37].

While there has been less systematic review of explicit diffusion-front metallogelation, these studies suggest that the diffusion of metal ions or ligands into a gelator-containing medium can be employed as a synthetic tool to control metallogel formation, particularly when inhomogeneous or layered structures are desired.

### 3.5. Sol–Gel-like Polymerization with Metal Nodes

**Figure 8:**
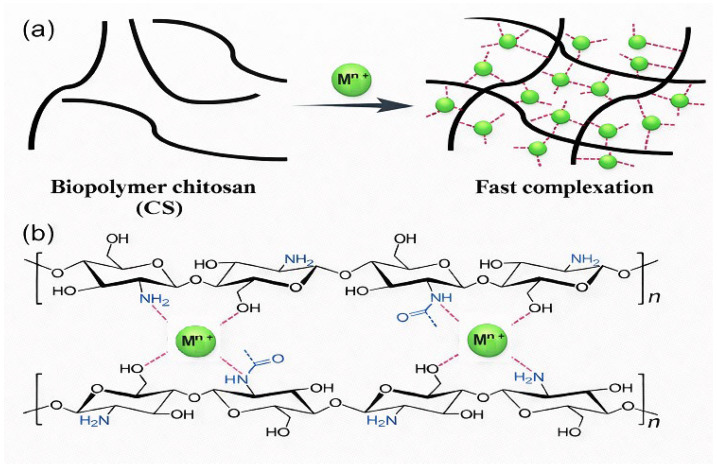
Schematic representation of metal–chitosan coordination leading to the rapid formation of polymer-network hydrogels in aqueous media (**a**). Chemical structures of chitosan and the resulting interlaced network architecture, driven by the complexation of metal ions with hydroxyl and amino moieties on the chitosan chains (**b**) [16].

Sol–gel polymerisation involving metal nodes combines the principles of inorganic sol–gel chemistry and metal–ligand coordination. In this process, metal ions or clusters act as cross-linking nodes for polymeric or oligomeric frameworks, typically through coordination with functional groups such as catechols, carboxylates, or phenolic oxygens. One reported sol–gel strategy involves pre-cross-linking plant polyphenols (such as tannic acid) with formaldehyde, followed by metal–ligand cross-linking with various metal ions (such as Co, Fe, Al, Ni, Cu, Zn, and Ce) to form metal–phenolic coordination spheres [96]. In 2015, Zhang et al. reported the formation of metallohydrogels through the supramolecular complexation of the natural biopolymer chitosan with a range of transition metal ions, including Ag(I), Cu(II), Co(II), Ni(II), Zn(II), Cd(II), and Pd(II) [16]. Owing to the strong and facile coordination of these metal ions with the amino and hydroxyl functionalities along the chitosan backbone, a series of transparent and mechanically stable metal hydrogels formed rapidly, typically within seconds, as illustrated in Figure 8a,b.

This study explicitly describes a formaldehyde-assisted metal–ligand cross-linking approach based on sol–gel chemistry, in which metal ions are incorporated as coordination nodes within an organic polyphenolic framework. A related strategy involves the crosslinking of polysaccharide hydrogels with metal ions. A recent review in the International Journal of Biological Macromolecules summarises how metal ions interact with anionic polysaccharide chains to form ionic polysaccharide hydrogels, thereby altering their physicochemical and rheological properties and enabling their use as versatile therapeutic carriers and tissue engineering materials [93].

In such systems, metal ions act as cross-linking centres, linking multiple polysaccharide chains and leading to gelation. These examples demonstrate that sol–gel-like polymerisation with metal nodes typically yields hybrid organic–inorganic networks, in which the polymer or polyphenol provides the organic scaffold, and the metal nodes introduce additional connectivity and functionality

### 3.6. Enzyme-Assisted Metallogelation

Enzyme-assisted metallogelation uses biocatalytic processes to encourage the formation of metal-containing gels in mild conditions. Although most reported enzyme-triggered gels are not explicitly labelled as metallogels, several studies demonstrate the enzyme-induced formation of organic–inorganic hydrogels involving metal ions. One article in the Journal of Materials Science describes the enzyme-induced mineralisation of hydrogels with amorphous calcium carbonate, in which an enzyme catalyses the formation of an inorganic CaCO_3_ phase within an organic network. This generates ultra-stiff, strong, and tough organic–inorganic double-network hydrogels. In this system, the enzyme controls the kinetics and spatial distribution of the metal-containing inorganic phase (based on Ca^2+^), resulting in hybrid gels with significantly improved mechanical properties. Complementary work on localised enzyme-assisted self-assembly shows that enzyme-triggered reactions can be used to generate supramolecular hydrogel coatings at specific locations on a substrate by catalysing the conversion of soluble precursors into self-assembling gelators [97,98].

Although that study focuses on peptide-based hydrogels with hyaluronic acid, it illustrates how enzymatic reactions can be used to achieve spatially confined gelation and tailored internal architecture. A concrete example of enzyme-triggered gelation that can be extended to metallogels is Alkaline phosphatase (ALP)-mediated dephosphorylation of aromatic peptide precursors. Several studies demonstrate that phosphorylated peptides remain soluble until ALP cleaves the phosphate group. The resulting dephosphorylated products then self-assemble into nanofibres and form hydrogels. For example, naphthyl-capped pentapeptides bearing phosphotyrosine (Nap–Phe–Phe–Gly–Glu–pTyr) undergo ALP-catalysed dephosphorylation and then assemble into nanofibrillar hydrogels. This study systematically compares the stereoisomers, dephosphorylation rates, proteolytic stability and cell compatibility of the resulting gels. Related systems using phosphoserine and phosphotyrosine in NapFF-based precursors demonstrate that phosphatase-catalysed dephosphorylation also produces supramolecular hydrogels, where the phosphorylation pattern controls the efficiency and extent of gelation [99,100].

Enzymes can also be integrated into metal-containing biopolymeric gels as metalloenzymes, providing an alternative approach to enzyme-assisted metallogelation. For instance, bioactive supports have been prepared using an–gelatin membranes crosslinked with glutaraldehyde and containing immobilised alkaline phosphatase and Mg^2+^ ions. These hybrid materials demonstrate preserved ALP activity and metal-ion content (Mg^2+^ and Zn^2+^), as well as promising cell viability and antibacterial performance [98].

In this case, the metal ions contribute to both the enzyme’s structure and function (ALP is a Zn/Mg metalloenzyme) and to the material’s overall bioinorganic character. While this system is not a classic supramolecular metallogel, it demonstrates how enzyme activity, metal ions and polymer networks can be combined within a single, gel-like structure. It also suggests design strategies in which enzyme activity resides within a metal-containing gel, which can modulate gel remodelling or facilitate further metal coordination. Overall, enzyme-assisted metallogelation can be considered a subset of enzyme-induced self-assembly and enzyme-driven hybrid gel formation. In this process, enzymes act as triggers, converting soluble precursors into self-assembling, metal-binding gelators or generating metal-containing inorganic phases. Metal ions or metal-containing phases provide structure and function, serving as coordination nodes, mineral phases or cofactors in metalloenzymes. Furthermore, spatial and temporal control can be achieved by localising enzyme activity, using enzyme gradients, or coupling enzymatic reactions with diffusion or light patterning [101,102].

### 3.7. Bio-Derived Metallogel Fabrication

**Figure 9:**
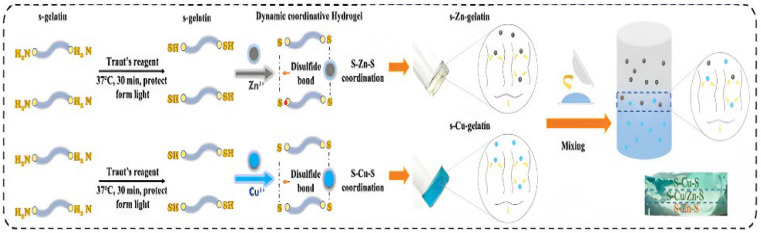
The principle and fabrication of the gradient bimetallic ion-based hydrogels and the operation diagram [103].

The fabrication of bio-derived metallogels employs naturally sourced biomolecules, such as polysaccharides, peptides, polyphenols, and proteins, as gelators or components of the gel matrix. These biomolecules are then coordinated with metal ions to obtain functional metallogels. A comprehensive review of metallogels for biomedical applications emphasises that natural ligands, including peptides, amino acids, nucleosides, and polyphenols, are increasingly being used as building blocks in metal-ligand coordination-based gels due to their biocompatibility and intrinsic functionality [12]. The fabrication principle of bimetallic ion–based hydrogels is illustrated in Figure 9.

Metal–phenolic systems based on tannic acid (TA) are a prototypical example. An article in Science Reports describes metallogels formed by mixing tannic acid with Ti(IV) ions to obtain TA–Ti(IV) gels; these gels can incorporate additional antimicrobial metal ions such as Fe(III), Cu(II), Zn(II), Co(II), and Ni(II) via co-gelation, leading to intelligent dressings for infected wounds with tunable metal release and strong antimicrobial activity [72]. More recent work on tannic-acid-based bio-metal–phenolic networks prepared with Cu(II), Zn(II), Bi(III), Ce(III), La(III), and Ti(IV) has shown that such bio-MOF or metal–phenolic materials can be hemocompatible, non-cytotoxic, antioxidant, and strongly antimicrobial, illustrating how bio-derived polyphenols and metal ions can be combined into functional hybrid materials [104]. A related study on sustainable metal–phenolic hybrid adsorbents reports double-crosslinked beads based on alginate and carboxymethyl cellulose with tannic acid-derived metal–phenolic frameworks, highlighting the potential of bio-derived metal–phenolic gels and beads as sustainable sorbents for ammonium removal [105]. In parallel, the polysaccharide hydrogel review mentioned above documents the extensive use of metal-ion-crosslinked natural polysaccharides (e.g., alginate and other anionic biopolymers) as hydrogels with tunable mechanical and delivery properties [94]. Combined with the biomedical metallogels review [12], these works show that bio-derived metallogels unify biocompatibility, biodegradability, and sustainability with metal-derived functionalities such as antimicrobial behaviour, drug-binding capacity, antioxidant properties, and catalytic or redox activity (Table 4).

Overall, the synthesis pathways discussed in this section demonstrate that coordination chemistry and metal–ligand self-assembly are particularly well-suited to applications requiring precise nanoscale control, dynamic responsiveness and tunable structure–function relationships. However, these approaches are sensitive to composition and processing conditions. Polymer-based, sol–gel-like and bio-derived metallogels are more robust, scalable and biocompatible, but have reduced structural programmability.

Nanoparticle-forming and in situ reduction approaches enable multifunctionality and enhanced catalytic or antimicrobial performance, but introduce additional challenges related to reproducibility, metal release and regulatory translation. Consequently, the optimal synthetic route depends on achieving the right balance between functional complexity, mechanical stability, scalability and application-driven safety requirements.

## 4. Limitations and Regulations

Despite their broad range of demonstrated applications, there are several interconnected challenges related to safety, sustainability, reproducibility and regulation that must be overcome before metallogels can be translated beyond laboratory-scale studies.

### 4.1. Scientific and Environmental Safety Perspective

Metallogels containing metal ions or nanostructured components are of concern due to their size- and surface-dependent reactivity, which can differ substantially from that of bulk materials. Peer-reviewed studies on nanomaterial toxicology demonstrate that the size of nanoparticles, their surface chemistry, their state of aggregation, and their metal composition can strongly influence biological interactions, oxidative stress, and bioaccumulation. This underscores the need for careful risk assessment [106]. These concerns are reflected in regulatory guidance, with both the U.S. Food and Drug Administration (FDA) and the European Medicines Agency (EMA) emphasising that nanotechnology-enabled materials require case-by-case evaluation as conventional toxicological assumptions may not apply [107,108].

### 4.2. Waste Management and Environmental Fate

Additional barriers to large-scale deployment include waste management and environmental fate. Scientific life cycle and particle flow analyses reveal that metal nanoparticles can be released during product use and disposal. Wastewater treatment and waste handling processes often fail to capture or transform these particles fully. Studies on nanosilver, for instance, suggest that dissipative applications could result in substantial environmental emissions and poorly understood long-term consequences [109]. Similarly, regulatory and policy documents emphasise the importance of considering end-of-life scenarios, environmental exposure pathways and recyclability in the early stages of material design, particularly for materials intended for widespread or consumer-facing applications [108].

### 4.3. Reproducibility and Scalability

Reproducibility and scalability further complicate practical implementation. From a materials science standpoint, supramolecular and coordination-driven metallogels are inherently sensitive to concentration, metal–ligand stoichiometry, solvent composition, and thermal or mechanical history. This sensitivity can lead to batch-to-batch variability in structure and mechanical performance, as widely documented for supramolecular polymer networks [110,111]. Regulatory frameworks reinforce this concern by stressing the need for clearly defined critical quality attributes, standardised characterisation protocols, and robust manufacturing controls to ensure consistent performance and safety [107,108].

### 4.4. Regulatory Classification and Approval Pathways

Regulatory classification and approval pathways remain challenging, particularly for biomedical and environmental applications. The combination of dynamic metal–ligand coordination, potential metal release, and nanoscale features complicates product classification and risk assessment. Regulatory guidance documents emphasise that early alignment with regulatory expectations, long-term stability studies, and comprehensive safety evaluations are essential for facilitating approval and responsible deployment [107,108]. Together, these scientific and regulatory considerations highlight that addressing limitations related to safety, sustainability, reproducibility, and compliance is as critical as demonstrating novel applications for the future development of metallogels.

## 5. Conclusions

Metallogels have evolved into a diverse and application-rich class of hybrid soft materials, characterised by the significant structural and functional contributions of metal ions, metal complexes, and metal-derived nanoparticles. As this review has demonstrated, integrating metal coordination chemistry with supramolecular assembly provides exceptional tunability in network architecture, dynamic behaviour and physicochemical properties—attributes that distinguish metallogels from purely organic hydrogels and organogels. The classification framework presented, based on matrix type, dominant metal interaction, and functional output, illustrates that metallogels can be constructed rationally from a wide range of gelators, including polymers, polyphenols, nanoparticles, peptides, and low-molecular-weight ligands. These gelators use distinct, yet often convergent, gelation mechanisms. The synthesis pathways summarised in Section 3 demonstrate that metallogels can be engineered through coordination-driven assembly, metal–ligand self-organisation, in situ nanoparticle formation, diffusion-regulated structuring, sol–gel-like polymerisation, enzyme-assisted triggering or bio-derived cross-linking. These methods provide control over fibre morphology, nanoscale ordering, mechanical properties, porosity, redox behaviour and responsiveness to chemical or physical stimuli. In terms of functionality, metallogels offer a unique convergence of soft-matter mechanics with metal-based catalytic, electronic, optical, antimicrobial and redox features. Catalytic metallogels benefit from accessible metal nodes or nanoparticulate domains that facilitate electrocatalysis, oxidative transformations and enzyme-mimetic activity. Conductive and metallo-electronic gels utilise metal-rich networks to enable charge transport, semiconducting behaviour and optoelectronic responses. Luminescent metallogels incorporating AIE chromophores, lanthanides or metal nanoclusters enable high-contrast sensing, imaging and anti-counterfeiting applications. Antimicrobial and biomedical metallogels demonstrate how controlled metal release, ROS generation, tissue-interactive mechanics and biocompatible matrices can support wound healing, infection control and regenerative medicine. Together, these examples emphasise how the interplay of supramolecular assembly and metal coordination enables multifunctionality that cannot be achieved in conventional gels.

Looking ahead, major opportunities in this field include developing predictive structure–function relationships that link metal identity, coordination geometry and network topology to macroscopic properties. In particular, establishing that relationships will be crucial for rational materials design, while the integration of metallogels into soft robotic and adaptive systems offers a promising route to exploit their intrinsic softness, responsiveness, and multifunctionality. Other opportunities include designing controllable multi-metal architectures and sustainable fabrication strategies, particularly those that employ bio-derived building blocks and green chemistry principles. In terms of applications, integrating metallogels into electronic skins, soft robotics, catalysis-on-gel platforms, on-tissue electronics and adaptive biomedical scaffolds will require a better grasp of how they behave under dynamic physiological, mechanical and electrochemical conditions. In this context, advances in operando spectroscopy, small-angle scattering techniques and multiscale modelling will be essential for mapping gelation pathways and guiding targeted material design. Overall, metallogels represent a rapidly evolving family of adaptive, multifunctional soft materials whose versatility arises from the synergy between organic self-assembly and metal-centred chemistry. As our understanding of their mechanisms and fabrication methods continues to improve, metallogels are set to transform catalysis, biomedicine, sensing, energy conversion and emerging soft technologies.

## Figures and Tables

**Table 1:** Classification of metallogels by matrix type.

Class	Primary Structural Component	Metal Role	Key Properties	Representative Applications	Refs.
MHs	Water-rich polymeric/supramolecular networks	Coordination cross-links	Self-healing, shear-thinning, stimuli-responsive	Drug delivery, wound healing, catalysis	[14,15,16,17,18]
MOGs	Metal ions + multidentate organic linkers	MOF/coordination-polymer-like nodes	Porosity, catalytic activity, tunable mechanics	Adsorption, catalysis, electrocatalysis	[11,16,17,19,20,21]
MPGs	Polyphenols (e.g., tannic acid)	Metal-ligand coordination (catechol/gallol)	Adhesive, antioxidant, antimicrobial	Coatings, drug delivery, wound healing	[22,23,24,25]
MNPGs	Metal nanoparticles	NP assembly, catalytic or plasmonic behaviour	Conductive, antimicrobial, catalytic	Sensing, catalysis, soft electronics	[26,27,28,29,30]
PMGs	Functionalised polymers	Reversible/permanent metal cross-links	Tunable viscoelasticity, self-healing	Soft electronics, sensors	[14,15,18]
Low-Molecular-Weight Metallogels (LMW-MGs)	Small ligands (<1500 Da)	Metal-triggered supramolecular assembly	Reversible gel–sol transitions, highly responsive	Catalysis, sensing, templating	[16,17,18,34]

**Table 2:** Metallogels classification by metal interaction type.

Interaction Type	Description	Advantages	Typical Metals/Systems	Refs.
Coordination-driven networks	Metal-ligand bonding forms extended networks	Predictable geometry, structural tunability	Coordination polymer gels, MOF-like gels	[14,16,17,35,36,37]
Discrete metal-ligand complexes	Supramolecular aggregation of metal-ligand complexes	High responsiveness & reversibility	Terpyridine complexes, Schiff-base complexes	[16,17,35]
Nanoparticle-forming metallogels	In situ or preformed NPs create 3D network	Catalytic, antimicrobial, plazmonic	Ag, Au, Pt, Fe_3_O_4_	[26,27,28,29,30,31,32,33],
Redox-active systems	Redox state controls assembly or transitions	Switchability, tunable optical/electronic properties	Ferrocene, Cu(I)/Cu(II), radical systems	[8,16,36,49,50,51]
Ionic cross-linking	Metal ions bridge biopolymer or polymer chains	Simple preparation, biocompatibility	Ca^2+^, Mg^2+^, Zn^2+^ systems	[52]

**Table 3:** Functional categories of metallogels.

Function	Mechanistic Origin	Key Characteristics	Example Applications	Refs.
Catalytic metallogels	Metal nodes act as reactive centres	High surface area, porosity, redox activity	Decontamination, OER/ORR electrocatalysis	[53,54,55,56,57,58]
Conductive/metallo-electronic gels	Charge transport via metal centres or NP domains	Semiconducting behaviour, diode characteristics	Soft electronics, sensors	[59,60,61,62,63,64,65,66]
Luminescent metallogels	Metal ions, AIE chromophores, or nanoclusters	AIE, tunable emission, sensing capability	Detection of nitroaromatics, anticounterfeiting	[53,67,68,69,70,71,72]
Antimicrobial & biomedical metallogels	ROS generation, controlled metal release	Antimicrobial, wound healing, tissue regeneration	Dressings, scaffolds, antibacterial coatings	[12,27,68,73,74,75]

**Table 4:** Summary of Metallogel Synthesis Pathways.

Synthesis Route	Key Principle	Advantages	Limitations	Representative Systems	Refs.
Coordination chemistry	Metal–ligand networks form gel	Robust, tunable	Crystallisation tendency	Coordination polymer gels	[35,37,85,86]
In situ reduction	Reduction of metal ions within gel matrix	NP dispersion, enhanced functionality	Requires reducing triggers	AgNP peptide gels	[26,27,89,90]
Metal–ligand self-assembly	Metal ions trigger LMWG assembly	Mild, reversible, stimuli-responsive	Sensitive to conditions	Cu^2+^-terpyridine gels	[32,39,66,93]
Diffusion-mediated	Diffusing ions create spatially patterned gels	Gradient control, structuring	Slow, less common	Patterned supramolecular hydrogels	[37,39]
Sol–gel-like polymerisation	Metal nodes cross-link polymer or polyphenol frameworks	Scalable, robust networks	Possible heterogeneity	TA-metal systems	[91,94]
Enzyme-assisted metallogelation	Enzymes produce gelators or inorganic phases	Biocompatible, spatial control	Enzyme sensitivity	ALP-triggered hydrogels	[95,96,97,98,99,100]
Bio-derived fabrication	Natural ligands + metal ions	Sustainable, biocompatible	Lower precision	TA-Ti(IV), alginate–metal gels	[12,73,93,103,104]

## Data Availability

No new data were generated in this review article. All studies and data reported are available publicly from the references cited.
